# Integrating cuproptosis- and ferroptosis-related gene signatures to predict prognosis, immunotherapy response, and drug sensitivity in patients with skin cutaneous melanoma

**DOI:** 10.3389/fimmu.2025.1742614

**Published:** 2026-01-14

**Authors:** Hua Dong, Ying Huang, Jie Shen, Zhaofeng Ding, Wei Wang, Yaping Li

**Affiliations:** 1 Department of Plastic Surgery, Southwest Hospital, Third Military Medical University (Army Medical University), Chongqing, China; 2 Oncology Department, Xinqiao Hospital, Army Medical University, Chongqing, China

**Keywords:** cuproptosis, drug sensitivity, ferroptosis, immunotherapy response, skin cutaneous melanoma

## Abstract

**Objective:**

Skin cutaneous melanoma (SKCM) is a highly aggressive malignancy originating from melanocytes, with a continuously rising global incidence. Developing strategies for early prevention and precise treatment remains a major challenge in oncology. Notably, advances in immunotherapy have brought new hope to SKCM patients. Increasing evidence suggests that various forms of regulated cell death, particularly cuproptosis and ferroptosis, can modulate the tumor microenvironment (TME) by inducing the death of both tumor and immune cells, thereby influencing the efficacy of immunotherapy. Consequently, there is a critical need to establish methods for early diagnosis and to develop reliable prognostic models prognostic models based on immune-related biomarkers.

**Methods:**

We integrated RNA sequencing data and corresponding clinical information from SKCM patients obtained from The Cancer Genome Atlas (TCGA) and the Gene Expression Omnibus (GEO) databases. Using single-cell transcriptomic data from the GSE72056 dataset, we analyzed the expression patterns of cuproptosis-ferroptosis-related genes (CFRGs) in SKCM and their enrichment in immune cell subsets. Key CFRG features were screened using machine learning algorithms to construct a prognostic risk-scoring model, which demonstrated robust predictive performance across multiple independent cohorts. Furthermore, we explored the associations between core CFRGs and patient survival outcomes, immunotherapy response, and drug sensitivity in SKCM.

**Results:**

We identified 10 key genes that were significantly associated with SKCM survival and successfully constructed a machine learning-based prognostic prediction model. This model showed strong predictive performance and demonstrated superior accuracy compared with existing prognostic models, as supported by cross-cohort and cross-cancer validation. Four key genes (IFNG, PTPN6, SLC38A1, and SOCS1) were further identified through association analyses with clinical phenotypes and showed significant correlations with clinical characteristics. Distinct immune cell infiltration patterns were observed between high- and low-risk groups stratified by these genes, indicating marked heterogeneity within the TME. This heterogeneity may directly influence patient responses to immunotherapy. Additionally, molecular docking analyses identified several potential therapeutic compounds, among which selumetinib demonstrated strong binding affinity to the target proteins IFNG, PTPN6, and SOCS1, suggesting a potential therapeutic strategy for advanced SKCM.

**Conclusions:**

IFNG, PTPN6, SLC38A1, and SOCS1 may serve as potential biomarkers of poor prognosis in SKCM patients. These genes demonstrate predictive value for immunotherapy response and drug sensitivity, particularly indicating susceptibility to selumetinib treatment, and therefore show substantial potential for clinical translation.

## 1 Introduction

Skin cutaneous melanoma (SKCM) is a malignant tumor originating from melanocytes in the epidermis. Although it primarily affects the skin, SKCM can also impact mucosal sites, including the genital tract, nasal passages, and oral cavity (1). In recent years, the global incidence of SKCM has continued to increase, with a growth rate remarkably higher than that observed for most other malignancies (1). Due to its aggressive nature and high metastatic potential, SKCM has become one of the leading causes of skin cancer–related deaths (2). Poor prognosis in SKCM is closely linked to its propensity for distant metastasis. Although numerous studies have focused on identifying high-risk SKCM patients and elucidating mechanisms that influence patient survival (3, 4), reliable biomarkers and accurate risk assessment systems to guide diagnosis and treatment remain limited.

Cell death plays a central role in maintaining homeostasis and mediating stress responses. Among these processes, regulated cell death is particularly important in tumor initiation and progression and has emerged as a promising direction for anticancer drug development (5, 6). Regulated cell death can influence tumor progression by modulating the immunogenicity of TME (6). Notably, cuproptosis and ferroptosis are two recently highlighted forms of regulated cell death that provide new perspectives for overcoming therapeutic challenges in SKCM through their interactions with the TME (7). Cuproptosis, a newly identified programmed cell death pathway, is triggered by copper ions binding to proteins involved in the mitochondrial tricarboxylic acid cycle (8) and has been associated with prognosis and treatment response in multiple cancers (9). Ferroptosis is an iron-dependent, non-apoptotic form of cell death characterized by lipid peroxide accumulation leading to membrane damage (10), and has demonstrated regulatory potential in SKCM (11) as well as other tumor types. Although both cuproptosis and ferroptosis contribute to SKCM pathogenesis, and several studies have attempted to integrate genes related to these processes to predict cancer prognosis and immunotherapy response (12), their combined mechanisms in SKCM and their systematic predictive value for the immune microenvironment and clinical outcomes remain poorly understood. Therefore, this study aims to construct a combined signature model based on CFRGsto predict immune features and clinical prognosis in SKCM patients, thereby providing new insights into precise classification and personalized treatment. In recent years, immune checkpoint inhibitors (ICIs), including anti-CTLA-4, anti-PD-1, and anti-PD-L1 antibodies, have achieved notable progress in SKCM treatment (13, 14). However, their overall clinical efficacy remains suboptimal, suggesting that immunoregulatory mechanisms or key molecular factors within the TME may constrain effective immune responses. Thus, identifying prognostic molecular markers and exploring their underlying mechanisms in SKCM is of considerable importance. Although ICIs have improved survival across various cancers, including SKCM, their clinical application continues to face challenges such as immune-related adverse events (irAEs) and therapeutic resistance (15). The TME represents a complex ecosystem composed of tumor cells, immune cells, stromal components, and extracellular elements (16), and is closely associated with tumor metastasis, treatment response, and immunosuppression (17, 18). TME characteristics have proven valuable for predicting immunotherapy response and patient survival (19, 20). Nevertheless, the clinical utility of established biomarkers, such as BRAF mutations (21) and PD-L1 expression (22) is limited by detection constraints and TME heterogeneity, underscoring the urgent need to identify novel biomarkers capable of integrating multidimensional TME information.

Based on this background, we systematically applied weighted gene co-expression network analysis (WGCNA) and the ESTIMATE algorithm to identify gene modules associated with SKCM development, followed by machine learning approaches to identify 10 core genes. Bioinformatics analyses and experimental validation revealed that IFNG, PTPN6, SLC38A1, and SOCS1 are closely associated with patient survival and immune regulation. These genes are highly expressed in SKCM and significantly correlate with tumor invasion, metastasis, and T-cell infiltration, particularly CD4^+^ T cells and immune checkpoint molecules such as CD274 and CD276. Molecular docking analyses further indicated that tumor cells with elevated expression of these genes may be more sensitive to targeted therapies, especially selumetinib. Together, these findings reveal the important roles of IFNG, PTPN6, SLC38A1, and SOCS1 in TME regulation and SKCM progression, highlighting their potential as prognostic biomarkers and therapeutic targets.

## 2 Materials and methods

### 2.1 Source of original data

Transcriptomic data (log2(counts+1) values) from 23 normal and 459 tumor tissues of cutaneous melanoma were obtained from The Cancer Genome Atlas (TCGA) via the UCSC Xena database (http://xena.ucsc.edu/). Clinical data were also retrieved for subsequent survival analysis and univariate and multivariate Cox regression analyses, including patient survival status, age, T stage, N stage, M stage, and tumor grade. In addition, Genotype-Tissue Expression (GTEx) data were downloaded from the same source to compare the differential expression of model genes between TCGA-SKCM tumor tissues and GTEx normal tissues.

The Gene Expression Omnibus (GEO, https://www.ncbi.nlm.nih.gov/geo/) is a public repository primarily containing high-throughput gene expression data and is widely used in cancer research. From GEO, the datasets GSE65904 (N = 214) and GSE54467 (N = 79) were obtained, from which melanoma patient survival information was extracted for external validation of the model developed from TCGA. Additionally, the GSE72056 melanoma single-cell RNA-seq dataset was acquired to analyze immune infiltration of core genes.

To evaluate the expression of model genes at the protein level, immunohistochemistry (IHC) images of the corresponding genes were retrieved from the Human Protein Atlas (HPA; https://www.proteinatlas.org/). These images enabled comparison of protein expression between melanoma tissues and normal skin samples.

We obtained genes related to cuproptosis cracking from previous studies conducted by others. Among them, there are 13 genes identified by Tsvetkov and other researchers, as well as 14 genes reported by Wang and other researchers. This article is also from inside the FerrDb database (https://www.zhounan.org/ferrdb/), and with ferroptosis. Subsequently, in this paper, the genes related to cuproptosis and ferroptosis were integrated to construct a brand-new joint marker, which is called CFRGs.

### 2.2 Weighted gene coexpression network analysis

We wanted to identify the genes that could predict the response to melanoma immunotherapy, so we utilized the ESTIMATE algorithm, version 1.0.13 (https://bioinformatics.mdanderson.org/estimate/index.Html). We used this algorithm to calculate matrix evaluation, immune, and ESTIMATE score, at the same time, to calculate the tumor purity of each sample. Rely on such calculations to assess the degree of immune infiltration. WGCNA was conducted using the gene expression profiles of the top 5000 most variable genes in the TCGA-SKCM cohort, with a soft threshold set to 0.9. Subsequently, the correlation between gene significance (GS) and module membership (MM) values was calculated to screen for subtype-representative modules. Modules were identified as subtype-representative based on two criteria: a significant correlation between GS and MM (p < 0.01), and a correlation coefficient > 0.8 between module eigengenes (ME) and external traits. Hub genes within specific modules were selected using the screening criteria of “GS > 0.2 and MM > 0.8”. We used the WGCNA algorithm to detect the key gene modules with the strongest correlation to the ESTIMATE score. Then, we isolated the genes in the core module from these key gene modules. Subsequently, we analyzed the genes in these modules and those related to cuproptosis and ferroptosis residues. That is, the overlap between the predefined features of CFRGs. Through such an analysis, the definition of the immune-related CFRGs set in melanoma was determined.

### 2.3 Construction and validation of the CFRGs prognostic risk model

To identify genes associated with the cuproptosis-ferroptosis combined signature (CFRGs) in melanoma, we first acquired expression matrices and corresponding survival data for immune-related CFRGs from three cutaneous melanoma datasets: TCGA-SKCM, GSE65904, and GSE54467. Predictive models were constructed using these genes, with TCGA-SKCM serving as the training set and GSE65904 and GSE54467 as external validation sets.

We integrated ten machine learning algorithms: Elastic Net (Enet), Ridge Regression, Cox Partial Least Squares Regression (plsRcox), Stepwise Cox Regression, Lasso, Survival Support Vector Machine (survival-SVM), Random Survival Forest (RSF), Supervised Principal Components (SuperPC), CoxBoost, and Generalized Boosted Regression Modeling (GBM). A total of 101 algorithm combinations were evaluated using 10-fold cross-validation within the TCGA-SKCM training cohort for feature selection and model construction. The predictive performance of each model was assessed using the concordance index (C-index) across both training and validation sets. The models were ranked according to their average C-index, and the top five performing approaches—StepCox[both] + SuperPC, StepCox[backward] + SuperPC, Lasso + SuperPC, CoxBoost + SuperPC, and survival-SVM—were selected for subsequent analysis. The Lasso + SuperPC algorithm was ultimately chosen as the final model. Ten genes were identified: ALOX5, NOX4, SLC38A1, IFNG, PTPN6, IL6, TLR4, CYBB, SOCS1, and FLT3. Using this algorithm, a risk score was computed for each patient based on the expression of these genes. Then, the patients are divided into high-risk and low-risk groups according to the median risk score. Subsequently, the survival differences between these groups are evaluated in the training group and the test group. To verify this model, this paper compared its C-index with the previously established prognostic models. By means of Cox regression analysis, nomogram construction, and decision curve analysis, the clinical application value of the risk model was evaluated.

### 2.4 Functional enrichment analysis

In this paper, the R software packages “clusterProfiler” and “enrichment” were used to conduct enrichment analyses of KEGG (Kyoto Encyclopedia of Genes and Genomes) and GO (Gene Ontology) enrichment analyses. GO analysis was mainly performed from three dimensions: cellular component (CC), molecular function (MF), and biological process (BP). KEGG analysis was conducted to explore disease-related pathways associated with the genes, and the most representative results were finally filtered with the criterion of *p*.adjust < 0.05. The Gene Set Enrichment Analysis (GSEA) software package was used to predict the potential signaling pathways involved in IFNG, PTPN6, SLC38A1, and SOCS1, so as to further investigate the biological functions of these genes in SKCM. The criteria for significantly enriched pathways were a normalized *p* value < 0.05 and an absolute value of the normalized enrichment score (NES) > 1.5.

### 2.5 Single-cell transcriptomics and immune infiltration analysis

First, a standardized analytical workflow was adopted to exclude low-quality cells identified by the criteria of nFeature_RNA < 300 and percent.mt ≥ 20. Subsequently, the data were normalized using the LogNormalize method. The FindVariableFeatures algorithm was applied to identify 2000 highly variable feature genes. Additionally, the “RunPCA” function was used to perform Principal Component Analysis (PCA) on the highly variable genes. We further employed t-Distributed Stochastic Neighbor Embedding (t-SNE), a widely used manifold learning technique, to perform dimensionality reduction and visualization of high-dimensional single-cell RNA sequencing (scRNA-seq) data, followed by cell clustering analysis. Based on the clustering results, the expression levels of specific marker genes in each cell cluster were calculated, and each cell cluster was annotated accordingly. Subsequently, the cell clusters containing target genes were further analyzed. Cell-cell communication networks among all cell types were inferred using the CellChat package based on scRNA-seq data, with interactions considered significant at *p* < 0.05.

Mutation data of SKCM was retrieved from the cBioPortal database, and the “maftools package” was used to visualize the gene mutation information in the form of a waterfall plot, so as to evaluate the mutation characteristics of different CFRG-related risk groups. To explore the relationship between immune cell infiltration and CFRGs, the CIBERSORT algorithm (https://cibersort.stanford.edu/) was employed to assess the level of immune cell infiltration in TCGA-SKCM samples by deconvolving the expression matrix of 22 human immune cell subtypes. Cross-validation was performed using the xCELL and ImmuCellAI algorithms. xCELL is an ssGSEA-based algorithm built on marker gene sets, which can calculate the abundance of 64 cell types, including adaptive and innate immune cells, hematopoietic stem cells, epithelial cells, and extracellular matrix cells (encompassing non-immune cell types). The ImmuCellAI database is a tool for estimating the abundance of 24 immune cell types, consisting of 18 T cell subtypes and 6 other immune cell types.

Additionally, Tumor Immune Dysfunction and Exclusion (TIDE) scores were retrieved from the TIDE online platform (http://tide.dfci.harvard.edu/) to compare immunotherapy responses between high- and low-CFRGs risk groups.

### 2.6 Molecular docking

To evaluate the correlation between high and low-expression groups of the core CFRGs genes in cutaneous melanoma and the drug Selumetinib, the three-dimensional (3D) structure of Selumetinib was obtained from PubChem (https://pubchem.ncbi.nlm.nih.gov/). The receptor proteins corresponding to the protein products of these core CFRGs were retrieved from the RCSB Protein Data Bank (PDB, http://www.rcsb.org/). Finally, CB-DOCK2 (https://cadd.labshare.cn/cb-dock2/) was used to predict and calculate the binding affinity between the candidate compounds and the target proteins.

### 2.7 Cell culture and transfection

In our research, the human malignant melanoma cell line A375 was obtained from WuChuan Bio. These cells were cultured in RPMI-1640 medium supplemented with 10% fetal bovine serum and 1% penicillin-streptomycin in an atmosphere containing 5% CO_2_ at 37°C. After the cells have reached approximately 70% confluence, we transfect the cells using siRNA targeting IFNG, PTPN6, SLC38A1, and SOCS1 according to the manufacturer’s instructions. After 72 hours, Western blot and reverse transcription quantitative polymerase chain reaction were used to confirm the efficiency of transfection.

### 2.8 Western blotting analysis

Total protein was extracted using a protein extraction kit and quantified with a BCA assay kit (B6167; US EVERBRIGHT). Protein was separated by SDS-PAGE (20230417;Solarbio) and transferred to PVDF membranes. After blocking with 5% skim milk, the membranes were incubated overnight at 4°C with the following primary antibodies:anti-IFNG(15365-1-AP;Proteintech),anti-PTPN6(#3759;Cell Signaling),anti-SLC38A1(12039-1-AP;Proteintech),anti-SOCS1(#68631;Cell Signaling), and anti-β-actin (66009-1-Ig;Proteintech).Subsequently, the membranes were incubated with an HRP-conjugated secondary antibody (bs-40296G-HRP; BIOSS; ab6721;abcam) for 2 hours at 37°C. Protein bands were visualized using an ECL chemiluminescent substrate (abs920;absin) and quantified with a BIO-RAD imaging system.

### 2.9 RT-qPCR

In this paper, we used reverse transcription quantitative polymerase chain reaction to detect the expression level of mRNA. Total RNA was extracted from cells using TRIzol reagent. The extracted total RNA was reverse transcribed into cDNA using a reverse transcription kit (R2020S; US EVERBRIGHT). Subsequently, the cDNA was amplified using the Bio-Rad CFX96 Real-Time PCR system. The primer sequences used were as follows.

β-ACTIN

Forward Primer: CATGTACGTTGCTATCCAGGC

Reverse Primer: CTCCTTAATGTCACGCACGAT

IFNG

Forward Primer: TCGGTAACTGACTTGAATGTCCA

Reverse Primer: TCGCTTCCCTGTTTTAGCTGC

PTPN6

Forward Primer: GGAGAAGTTTGCGACTCTGAC

Reverse Primer: GCGGGTACTTGAGGTGGATG

SLC38A1

Forward Primer: TGACAGTGCCCGAGGATGATA

Reverse Primer: AGACATGCCTAAGGAGGTTGTA

SOCS1

Forward Primer: TTTTCGCCCTTAGCGTGAAGA

Reverse Primer: GAGGCAGTCGAAGCTCTCG

### 2.10 CCK-8 proliferation assay

Logarithmically growing wild-type A375 cells and IFNG-, PTPN6-, SLC38A1- and SOCS1-knockdown cell lines were collected and prepared into single-cell suspensions with a concentration of 1×10^4^ cells/mL. Subsequently, 100μl of the cell suspension was seeded into each well of a 96-well plate (3 replicate wells per group). After cell adhesion, 10μl of CCK-8 reagent was added to each well at predetermined time points (0 h, 24 h, 48 h, 72 h), followed by incubation at 37°C in a 5% CO_2_ incubator for 2 hours in the dark. The absorbance (OD value) of each well was measured at a wavelength of 450 nm using a microplate reader.

### 2.11 Transwell invasion assay

A Matrigel layer was coated onto the upper chamber of a Transwell insert (containing a polycarbonate membrane) and polymerized at 37°C for 3 hours to model the extracellular matrix. Cells in serum-free medium were then seeded into the upper chamber, with the lower chamber containing a complete medium with chemoattractants. Following 24 hours of incubation, the cells on the membrane were fixed, stained with hematoxylin and eosin, and the membrane was removed and resin-mounted. Cell invasion was quantified by counting the number of cells that had migrated through the membrane under a 20× objective.

### 2.12 Cell scratch assay

Cells were seeded in 6-well plates at a density of 5×10^5^ cells per well to ensure the formation of a confluent monolayer by the following day. After 8 hours of culture, a sterile pipette tip was used to create a straight scratch in the cell monolayer, simulating a wound. Images of the scratch were immediately captured at a 4× magnification to establish the baseline (recorded as the 0-hour width). The plates were then returned to the incubator, and the same areas were re-imaged after 24 hours. Cell migration was assessed by measuring the changes in the scratch width using ImageJ software.

### 2.13 Statistical methods

In this study, the Wilcoxon rank-sum test was used to analyze the differential expression between SKCM cancer tissues from the TCGA database and normal tissues from the GTEx database. The same method was employed to compare differences in immune infiltration levels among different gene groups or risk groups. Univariate and multivariate Cox regression analyses were performed to evaluate the impact of risk scores and selected clinical factors on prognosis. Pearson correlation analysis was used to assess the correlations between the genes involved in this study. The magnitude of the association between hub genes and immune cells was determined using the Mantel test. All scatter plots, histograms, and heat maps were generated using GraphPad Prism 8.4.3 and the ggplot2 package in R. Present the data in graphical form with these tools. Statistical significance is defined as: ns, not significant, *p<0.05, **p<0.01, ***p<0.001, to determine whether the differences in the data are meaningful.

## 3 Figures

### 3.1 Acquisition of core module genes

**Figure 1:**
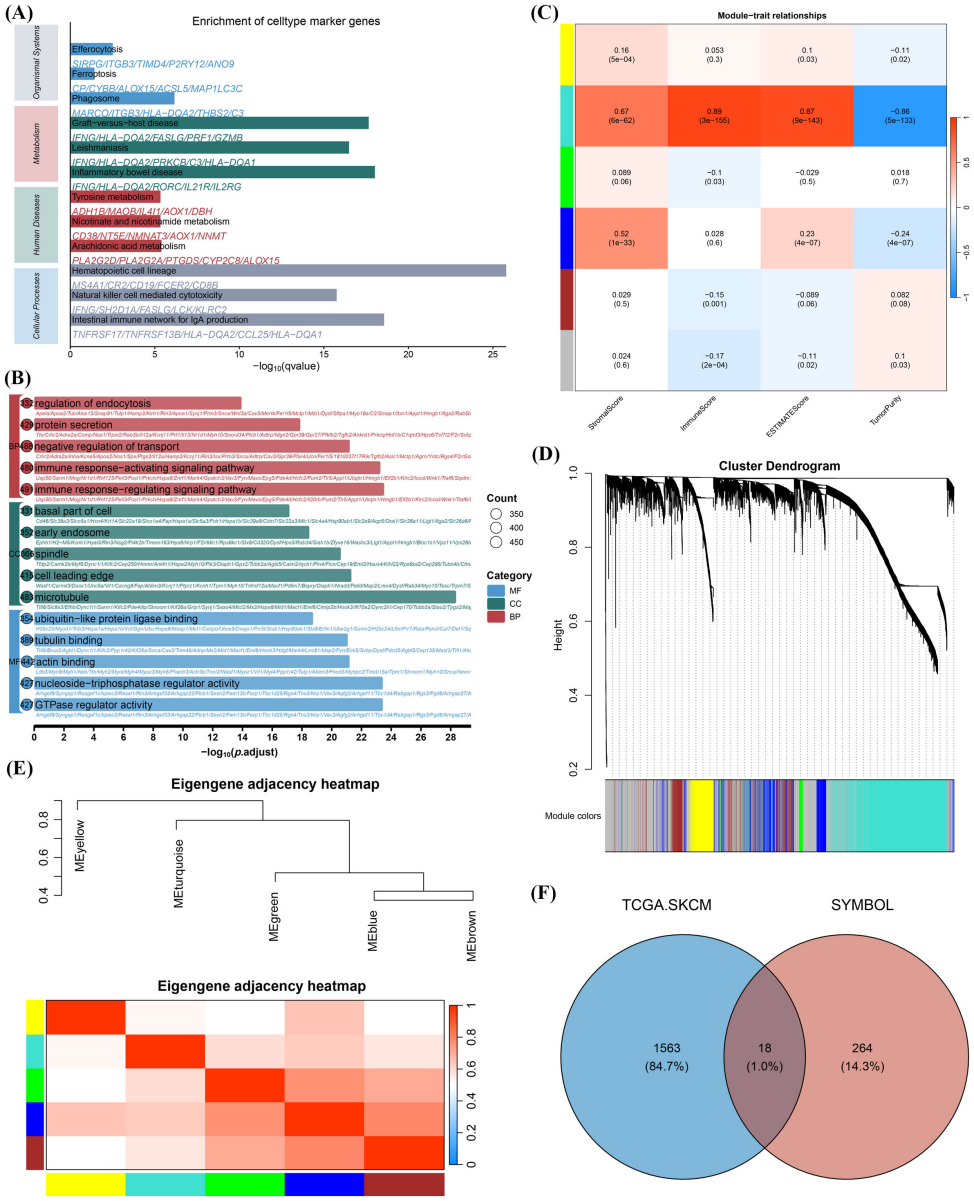
Identification of immune-related CFRGs. **(A)** KEGG enrichment analysis of genes in the red module. **(B)** GO enrichment analysis of genes in the red module. **(C)** Heatmap of module–trait relationships. **(D)** Clustering dendrogram of genes based on expression profiles. **(E)** Adjacency heatmap of feature genes. **(F)** Venn diagram showing the overlap between red module genes and CFRGs.

To identify CFRGs associated with melanoma immunity, we incorporated the top 5000 most expressed genes from the SKCM gene expression matrix into a weighted gene co-expression network analysis (WGCNA). The pickSoftThreshold function in the WGCNA R package was used to automatically determine the soft thresholding power. Multiple gene modules were identified using dynamic tree-cutting, and closely related modules were merged with the mergeCloseModules function to obtain the final consensus modules (**Figures 1D, E**). Pearson correlation analysis was conducted to evaluate the association between each module and SKCM stromal score, immune score, ESTIMATE score, and tumor purity. The most relevant module, labeled “Turquoise”, containing 1581 immune-related genes (**Supplementary Table 1**), was selected for further analysis (**Figure 1C**). Subsequent GO enrichment analyses indicated that these module genes were primarily involved in regulating endocytosis, protein secretion, immune response-activating signal transduction, basal region of cells, and GTPase regulator activity (**Figure 1B**). KEGG analysis further revealed enrichment in pathways such as phagosome formation, phagocytosis, ferroptosis, and GTPase regulator activity (**Figure 1A**). Among these, 18 genes were identified as associated with the CFRGs’ signature (**Figure 1F**), suggesting that they should be investigated as a functionally cohesive group.

### 3.2 Comprehensive construction of a CFRGs-based prognostic model

**Figure 2:**
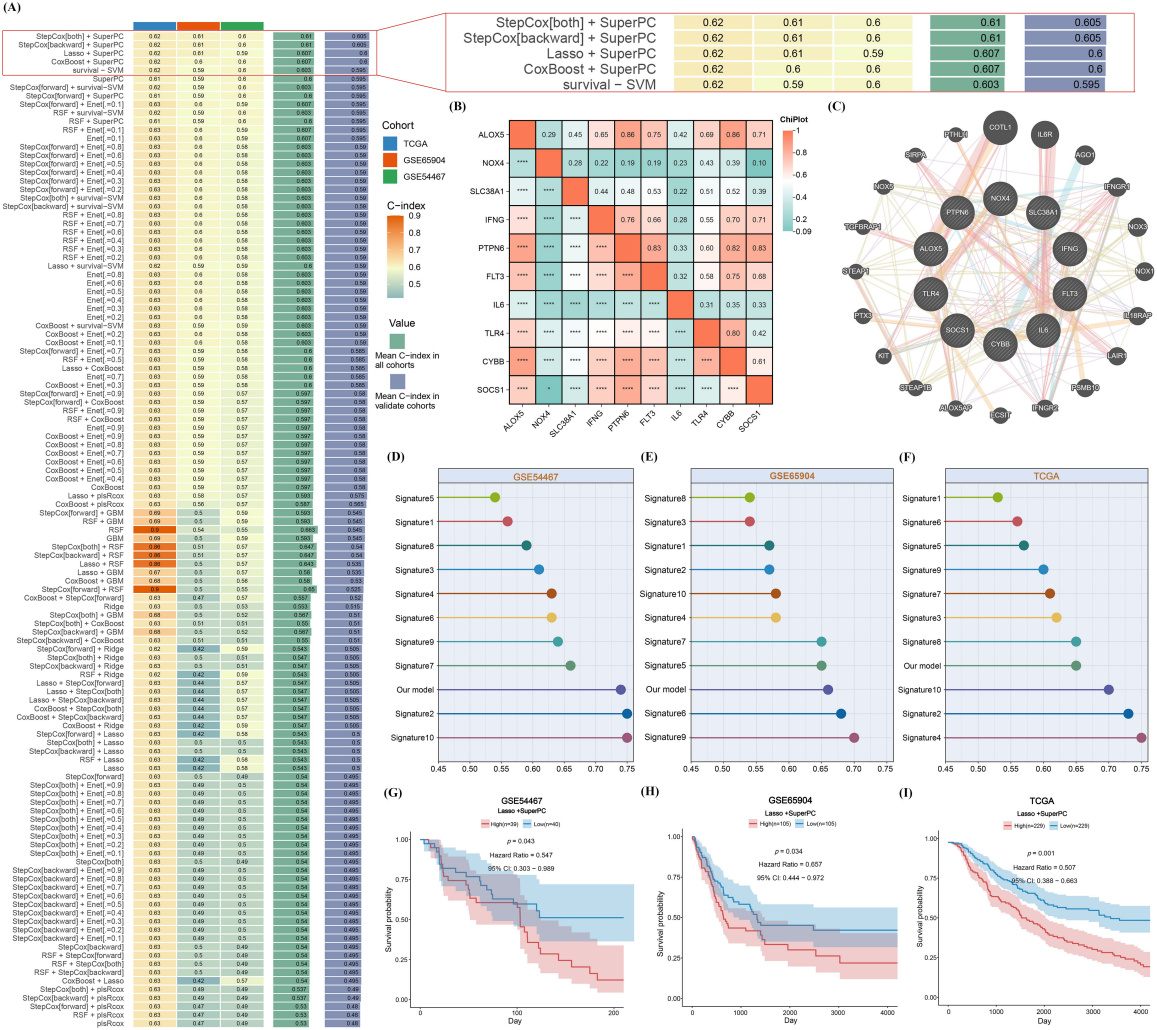
Construction of the CFRGs risk model. **(A)** C-index performance metrics of different algorithm combinations across multiple patient cohorts. **(B)** Heatmap of expression correlations among genes in the core prognostic gene set. **(C)** Protein-protein interaction (or co-expression) network of core prognostic genes. **(D–F)** Box plots comparing score distributions between the prognostic model proposed in this study and existing models across different validation cohorts. **(G–I)** Kaplan-Meier survival curves. Patients were stratified into high-risk (red) and low-risk (blue) groups based on model scores, showing significantly different survival probabilities across cohorts.

To further investigate cuproptosis- and ferroptosis-related genes (CFRGs), we implemented 101 machine learning algorithm combinations using the TCGA-SKCM dataset as the training cohort and GSE65904 and GSE54467 as validation cohorts (**Figure 2A**). Ultimately, the 5 most valuable algorithms were selected based on the C-index of the algorithms, and the “Lasso+SuperPC” algorithm was established as the final research method. A risk model was constructed based on this algorithm, and PCA was plotted (**Supplementary Figure S1A**). The results showed that most samples were statistically significant, and there was heterogeneity between the high and low risk groups. This approach identified the following ten model genes: ALOX5, NOX4, SLC38A1, IFNG, PTPN6, IL6, TLR4, CYBB, SOCS1, and FLT3(**Supplementary Table 2**). Correlation analysis revealed significant associations among these genes (**Figure 2B**), and interaction predictions from the GeneMANIA database indicated coordinated regulatory relationships (**Figure 2C**). Using the “Lasso + SuperPC” algorithm, a risk score based on the CFRGs’ signature was computed to stratify SKCM patients into high- and low-risk groups. Survival analysis demonstrated that high-risk patients had significantly poorer outcomes compared to low-risk patients in the TCGA-SKCM, GSE65904, and GSE54467 cohorts (**Figures 2G–I**). Moreover, the CFRGs-based model showed a higher C-index than other clinical features, confirming its superior predictive accuracy across all validation sets (**Figures 2D–F**).

### 3.3 Clinical value of the CFRGs-based model

**Figure 3:**
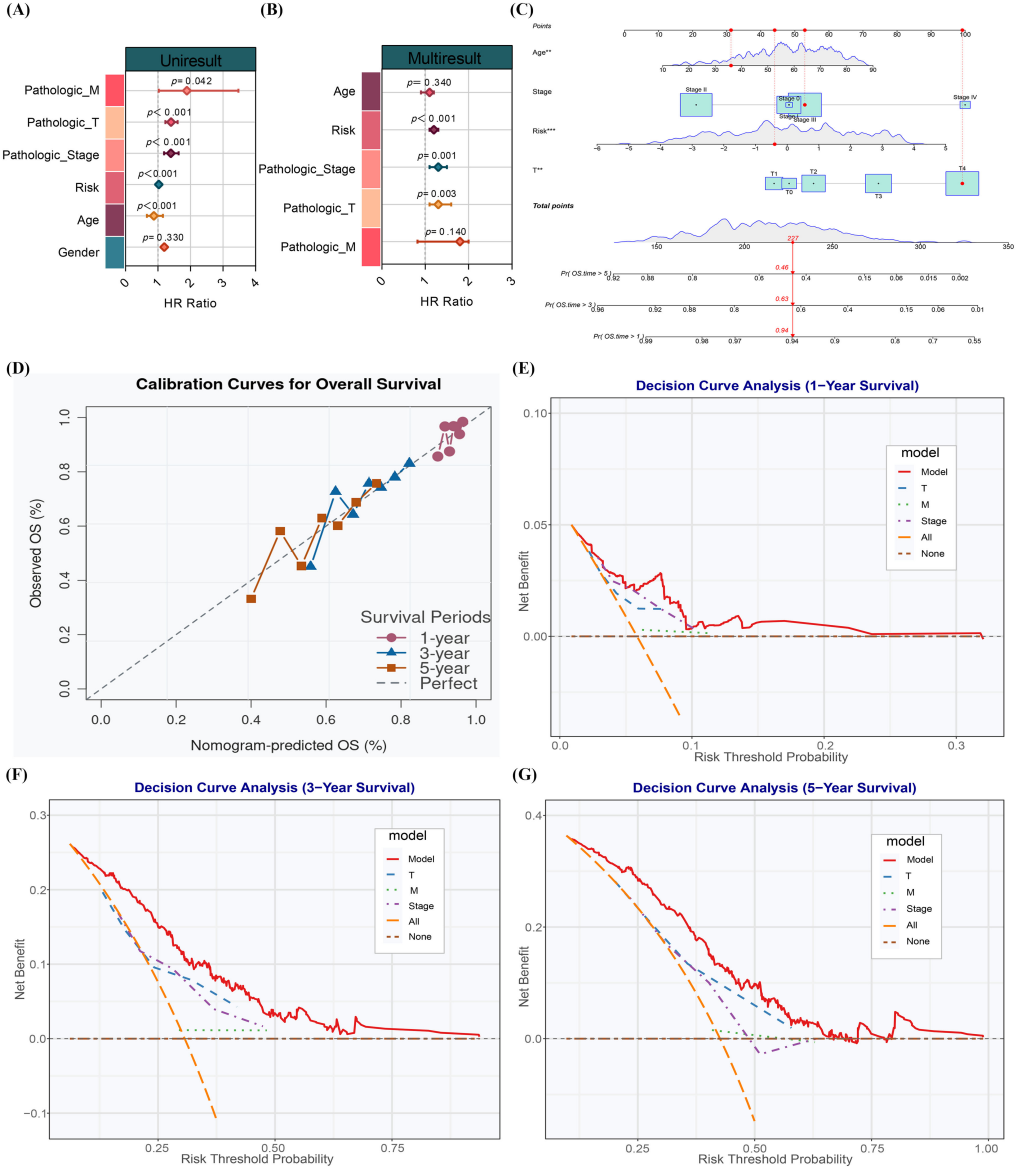
Performance evaluation of the CFRGs risk model. **(A, B)** Univariate and multivariate Cox regression analyses of the CFRGs risk score with different clinical variables. **(C)** Nomogram integrating the CFRGs risk score and clinical variables for calculating individual patient survival probability. **(D)** Calibration curves demonstrating good consistency between predicted and observed survival rates. **(E–G)** Decision curve analysis showing significant clinical net benefit of the model across different time points.

This article aims to assess the relationship between the CFRGs-based model and clinical staging. So, univariate and multivariate Cox regression analyses were conducted. The univariate analysis revealed that the CFRGs risk score, age, clinical T stage, M stage, and tumor grade all had a significant relationship with overall survival rate (**Figure 3A**). Among these, the CFRGs risk score, age, tumor grade, and M stage are crucial risk factors (HR > 1, p < 0.05). Multivariate analysis demonstrated that the CFRGs risk score is an independent prognostic factor for survival in SKCM patients (**Figure 3B**). To further clarify the relationship between this model and clinical progression, a nomogram was developed in this paper, which included age, T stage, tumor grade, and CFRGs’ risk score. Use it to predict the 1-year, 3-year, and 5-year survival rates of SKCM patients at different stages and grades (**Figure 3C**). The nomogram indicates that the survival rate decreases with age, the development of the T stage, and the increase in the CFRGs’ risk score. The calibration curve shows that there is a strong consistency between the predicted survival rate and the observed survival rate at 1 year, 3 years, and 5 years (**Figure 3D**), supporting the model’s reliability. The decision curve analysis shows that compared with other models, the CFRGs’ model can bring higher net benefits at time points of 1 year, 3 years, and 5 years (**Figures 3E–G**). These findings indicate that the prognostic model constructed by CFRGs-related differentially expressed genes has crucial clinical application value in the risk stratification and prognosis prediction of SKCM, and also demonstrate that the selected gene markers are scientifically effective.

### 3.4 Immune infiltration analysis of the CFRGs-based model

**Figure 4:**
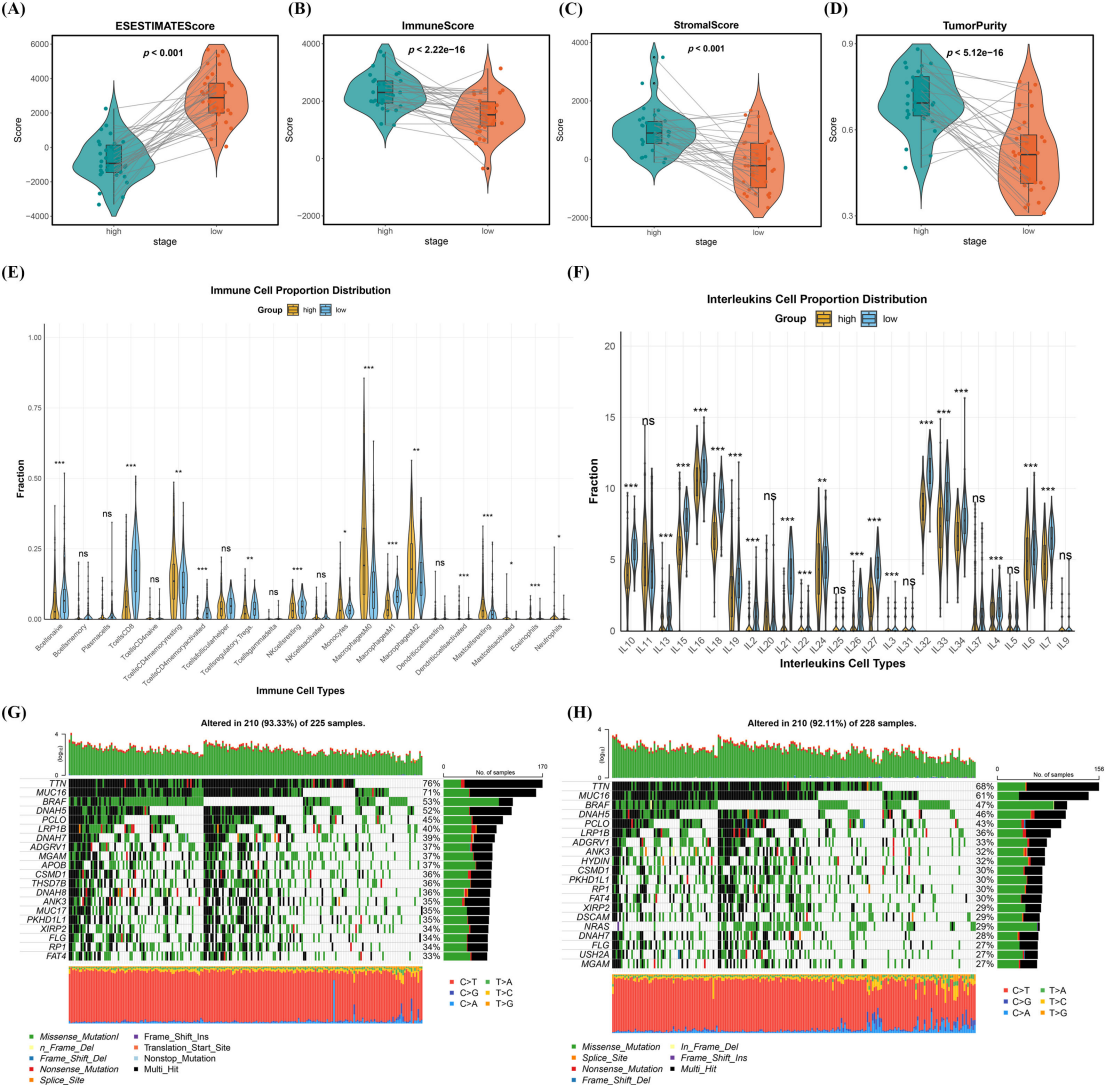
Evaluation of tumor immune microenvironment characteristics by the CFRGs risk model. **(A–D)** Violin plots (with box plots) comparing the distribution of ESTIMATE score, immune score, stromal score, and tumor purity between high-risk and low-risk CFRGS groups. **(E, F)** Box plots displaying the proportions of different immune cell infiltration and interleukin-associated cells in high-score versus low-score CFRGS groups. **(G, H)** Mutation analysis of high-risk and low-risk CFRGS groups, ns:not significant, *p< 0.05, **p< 0.01, ***p< 0.001.

Previous findings indicate a strong association between CFRGs’ expression and SKCM patient survival. Given the close relationship between cell death processes and antitumor immunity, we hypothesized that CFRGs may influence the SKCM immune microenvironment, thereby affecting clinical outcomes. To investigate this, we compared stromal, immune, and ESTIMATE scores (**Supplementary Table 3**), as well as tumor purity, between high- and low-CFRGs risk groups. All four parameters differed significantly between the groups (**Figures 4A–D**), with the high-risk group showing higher immune and stromal infiltration and greater malignant potential compared to the low-risk group. Using the CIBERSORT algorithm(**Supplementary Table 4**), we further analyzed the infiltration levels of 22 immune cell types and observed distinct patterns of CFRGs’ expression in B cells naïve, CD8+ T cells, CD4+ memory resting T cells, CD4+ memory activated T cells, regulatory T cells (Tregs), resting NK cells, and macrophages (M0, M1, M2) between the risk groups (**Figure 4E**). Cross-validation was performed using the XCELL algorithm (**Supplementary Figure S2A**) and ImmuCellAI algorithm (**Supplementary Figure S2B**) (**Supplementary Tables 5**, **6**). The results showed that CFRGs exhibited differential distribution across multiple immune cell types among different risk groups, particularly in CD8+ T cells, which further indicated that CFRGs are correlated with the immune microenvironment of SKCM. In addition, the researchers found that different CFRG-related risk groups showed differential expression in the vast majority of human leukocyte antigens (HLAs), including IL10, IL13, and IL15 (**Figure 4F**). Finally, the mutation status of different CFRG-related risk groups was analyzed based on SKCM mutation data retrieved from the cBioPortal database. The results showed that high-risk CFRGs were mainly associated with mutations in genes such as TTN, MUC16, BRAF, DNAH5, and PCLO (**Figure 4G**), while low-risk CFRGs were also primarily correlated with mutations in these same genes (**Figure 4H**). Although the key mutated genes were identical between the two groups, the high-risk CFRG group consistently exhibited a higher mutation frequency. In addition, TIDE and Dysfunction analyses were performed (**Supplementary Figures S3A, B**). The results revealed that the negative values in the high-risk group were significantly lower than those in the low-risk group, indicating low T-cell dysfunction and exclusion scores. It is further speculated that patients in the high-risk group may be more prone to immune escape.

### 3.5 Single-cell analysis of CFRGs expression patterns

**Figure 5:**
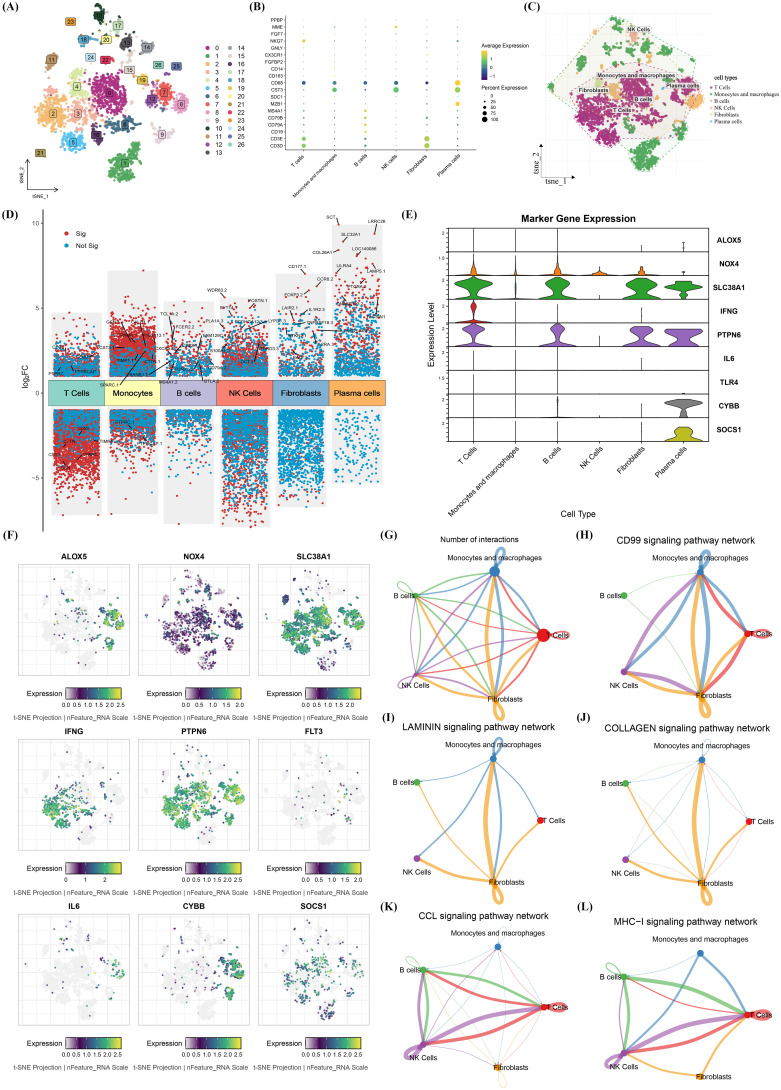
Single-cell sequencing analysis of immune-related CFRGSs. **(A)** Cell clustering based on high-dimensional data clearly displays the distribution of different cell subpopulations. **(B)** Bubble plot showing correlations between marker genes and cell clusters. **(C)** Cell distribution density map in t-SNE dimensional reduction space. **(D)** Volcano plot of differentially expressed genes across different cell groups. **(E)** Violin plots showing the distribution of 9 CFRGSs across different cell types. **(F)** t-SNE spatial distribution of 9 CFRGSs across different cells. **(G–L)** Key signaling pathway interactions between different cell subgroups (nodes) under the CellChat algorithm, and cellular interactions under different signaling pathways.

First, quality control, normalization, dimensionality reduction, and other preprocessing steps were performed on the single-cell RNA sequencing (scRNA-seq) dataset GSE72056 from the GEO database (**Supplementary Figures S4A–E**). Subsequently, clustering analysis was conducted on the standardized scRNA-seq data. The results showed that the SKCM samples in the GSE72056 dataset were clustered into 27 cell clusters (**Figure 5A**). The researchers annotated these clusters into 6 distinct cell types (**Figure 5C**) using marker genes from CellMarker 2.0 for differentially expressed cells across different clusters, namely T cells, macrophages, B cells, NK cells, fibroblasts, and plasma cells. Meanwhile, bubble plots were used to visualize the expression of marker genes for the 6 cell types (**Figure 5B**), and volcano plots were generated to display differentially expressed genes among different immune subsets (**Figure 5D**).We then examined the expression of the nine model genes across these cell types. The violin plot shows their expression distribution (**Figure 5E**). The t-SNE projection at the single-cell resolution level prominently presents their spatial expression patterns (**Figure 5F**). The results showed that genes such as IFNG, PTPN6, SLC38A1, SOCS1, and TLR4 were significantly expressed in these immune cell subsets, with primary associations with two major immune cell types: T cells and B cells. Further investigation into the intercellular communication network revealed significant connections among various cell types, particularly between B cells and T cells (**Figure 5G**). Additionally, we calculated the intercellular correlations within specific signaling pathways based on these pathways. We found that T cells and B cells exerted varying degrees of effects in processes such as the CD99 signaling pathway (**Figure 5H**), LAMININ signaling pathway (**Figure 5I**), COLLAGEN signaling pathway (**Figure 5J**), CCL signaling pathway (**Figure 5K**), and MHC-I signaling pathway (**Figure 5L**), among which T cells and B cells played crucial roles in the CCL signaling pathway and MHC-I signaling pathway. Through single-cell analysis, the researchers identified several more critical genes among these candidates, which were primarily associated with the two major immune cell types (B cells and T cells). This finding provides a novel insight for subsequent studies, which will focus on either B cells or T cells. Subsequently, correlation analysis was performed between CFRG-related risk scores and the signature markers of CD8+ T cells (**Supplementary Table 7**). The results demonstrated that CFRGs were negatively correlated with most signature markers of CD8+ T cells, suggesting that CFRGs may inhibit the activation of CD8+ T cells.

### 3.6 Enrichment analysis of SLC38A1, PTPN6, SOCS1, and IFNG

**Figure 6:**
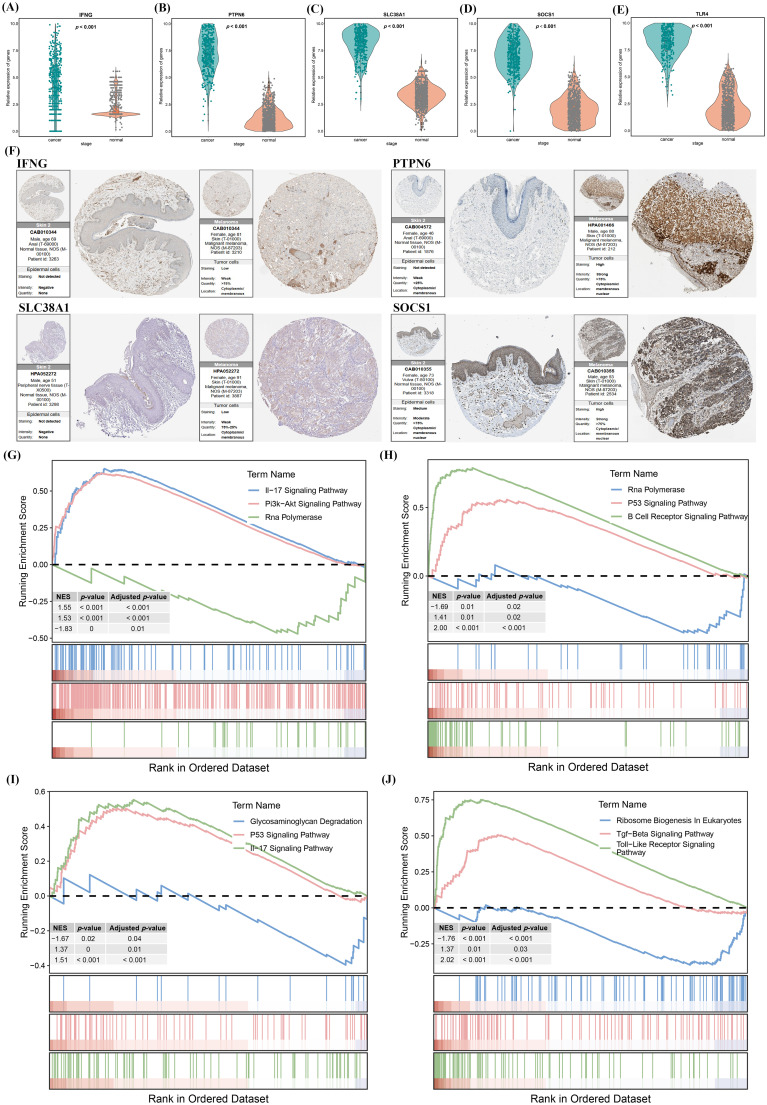
GSEA enrichment analysis of IFNG, PTPN6, SLC38A1, and SOCS1. **(A–E)** Differential analysis of IFNG, PTPN6, SLC38A1, SOCS1, and TLR4 based on TCGA and GTEx databases. **(F)** Differential protein expression of IFNG, PTPN6, SLC38A1, and SOCS1 in the HPA database. **(G–J)** GSEA enrichment analysis of IFNG, PTPN6, SLC38A1, and SOCS1.

Differential expression of the aforementioned 10 genes was compared between the TCGA database and the GTEx database. Among these genes, only 5 (IFNG, PTPN6, SLC38A1, SOCS1, and TLR4) were overexpressed (**Figures 6A–E**). To further explore their expression differences at the tissue level, comparisons were performed using the HPA database, and the results showed that IFNG, PTPN6, SLC38A1, and SOCS1 exhibited differential expression (**Figure 6F**). Therefore, this study identified IFNG, PTPN6, SLC38A1, and SOCS1 as immune-related CFRGs in SKCM, and subsequent research will focus on these genes. To further investigate their molecular functions, GSEA was performed for each of these four genes (**Supplementary Tables 8**-**11**). The results revealed that all four genes were associated with the PI3K-AKT signaling pathway, which has been previously reported to be closely involved in tumor migration and invasion (23–25). Partial enrichment results are presented as follows: IFNG was significantly correlated with signaling pathways such as the IL-17 signaling pathway,PI3K-AKT signaling pathway, and RNA polymerase (**Figure 6G**); PTPN6 mainly regulates the RNA polymerase, P53 signaling pathway, and B cell receptor signaling pathway (**Figure 6H**); SLC38A1 is primarily associated with the glycosaminoglycan degradation, P53 signaling pathway and IL-17 signaling pathway (**Figure 6I**); SOCS1 mainly exerts its functions in the Ribosome biogenesis In eukaryotes, TGF-b signaling pathway, and toll-like receptor signaling pathway (**Figure 6J**). GSEA results indicated that these hub genes may be involved in processes such as immunity and migration of melanoma, which were verified through experiments.

**Figure 7:**
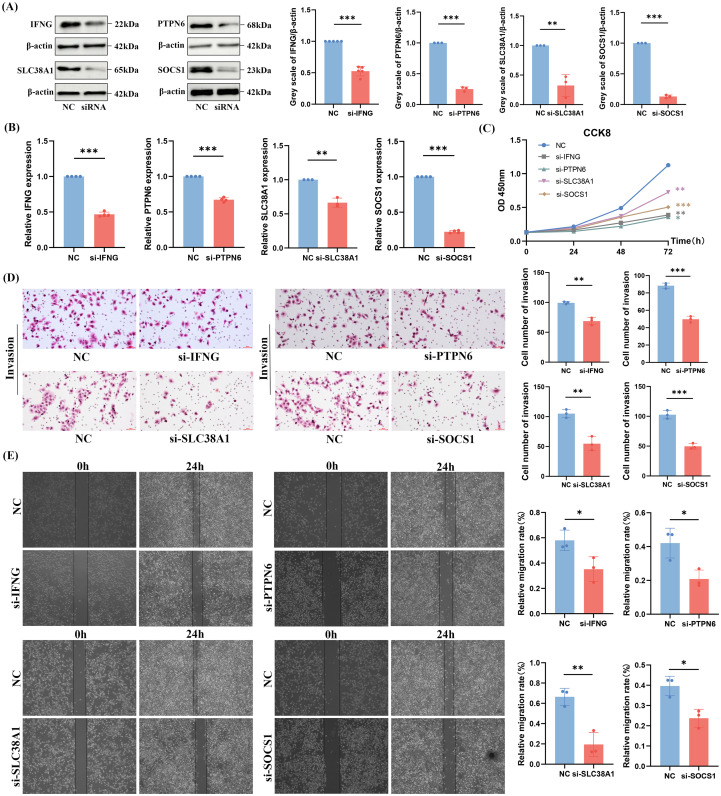
Proliferation, migration and invasion capabilities of A375 cells after knockdown of IFNG, PTPN6, SLC38A1 and SOCS1. **(A, B)** Protein and mRNA expression levels of IFNG, PTPN6, SLC38A1, and SOCS1 after siRNA transfection. **(C)** Proliferative capacity of A375 cells after knockdown of IFNG, PTPN6, SLC38A1, and SOCS1 via CCK8 assay. **(D)** Transwell invasion assay for detection of invasive capacity of A375 cells after knockdown of IFNG, PTPN6, SLC38A1 and SOCS1. **(E)** Wound healing assay for detection of migratory capacity of A375 cells after knockdown of IFNG, PTPN6, SLC38A1, and SOCS1, ns:not significant, *p< 0.05, **p< 0.01, ***p< 0.001.

First, the researchers constructed IFNG, PTPN6, SLC38A1, and SOCS1 knockdown cell lines, and verified their knockdown efficiency at the protein level (**Figure 7A**) and mRNA level (**Figure 7B**) using Western Blot and Reverse Transcription-Polymerase Chain Reaction assays, respectively. Subsequently, to further analyze the molecular functions of these genes,CCK-8 proliferation assay (**Figure 7C**), transwell invasion assays (**Figure 7D**) and wound healing assays (**Figure 7E**) were performed, followed by quantitative analysis. The results showed that the proliferation, invasion and migration abilities of cells with low expression of IFNG, PTPN6, SLC38A1, and SOCS1 were significantly attenuated, with statistical significance. The aforementioned studies indicated that IFNG, PTPN6, SLC38A1, and SOCS1 are of greater value in SKCM, as they are closely associated with the immune response of SKCM and can promote the proliferation, invasion and migration of SKCM cells. Both GSEA enrichment analysis and experimental results demonstrated that IFNG, PTPN6, SLC38A1, and SOCS1 may be involved in the proliferation, invasion, migration and immune infiltration of SKCM.

### 3.7 Immune infiltration characteristics of IFNG, PTPN6, SLC38A1, and SOCS1

**Figure 8:**
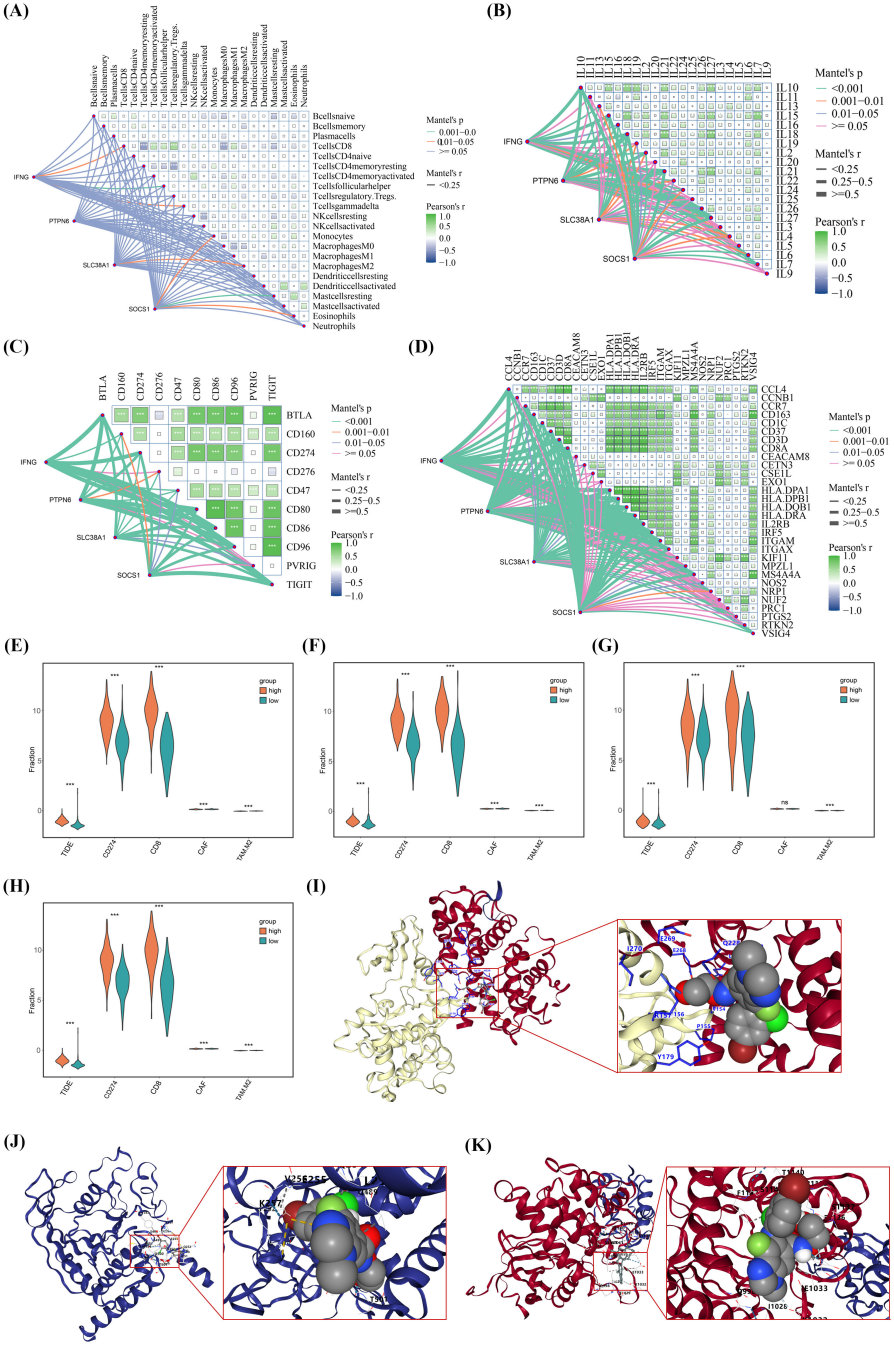
Immune and drug resistance analysis of IFNG, PTPN6, SLC38A1, and SOCS1. **(A–D)** Mantel’s test showed correlations of IFNG, PTPN6, SLC38A1, and SOCS1 with immune cells, leukocyte antigens, immune checkpoints, and immune cell markers, respectively. **(E-H)** Differential analysis of immunotherapy efficacy (TIDE, CD274, CD8, CAF, TAM.M2) between high and low expression groups of IFNG, PTPN6, SLC38A1, and SOCS1, respectively. **(I–K)** Molecular docking diagrams of IFNG, PTPN6, and SOCS1 with Selumetinib, ns:not significant, *p< 0.05, **p< 0.01, ***p< 0.001.

To further analyze the relationship between these genes and immune infiltration in SKCM, the researchers correlated these genes with immune cells, human leukocyte antigens (HLAs), immune checkpoint inhibitors, and signature genes of immune cells in SKCM, respectively. Mantel test results showed that IFNG, PTPN6, SLC38A1, and SOCS1 were significantly correlated with these immune-related markers (**Figures 8A–D**). Notably, the Mantel test again indicated that these genes were associated with CD8+ T cells and most HLAs, including IL10 and IL13. Among these, IL10 has been reported to be closely linked to melanoma (26, 27). This finding suggests that future research should focus on the relationships between the hub genes, CD8+ T cells, and IL10.Furthermore, differential expression of IFNG, PTPN6, SLC38A1, and SOCS1 in immune response-related contexts (including TIDE, CD274, CD8, CAF, and TAM.M2) was analyzed using the TIDE database (**Figures 8E–H**). The results showed that patients with high expression of IFNG, PTPN6, SLC38A1, and SOCS1 derived greater benefits from immunotherapy, indicating that these patients were more refractory to immunotherapy. Finally, the binding affinity between these genes and Selumetinib (a chemotherapeutic drug commonly used for SKCM) was evaluated using the CB-Dock2 database. The results revealed that the binding energy scores of IFNG, PTPN6, and SOCS1 with Selumetinib were all higher than -7 kcal/mol (**Supplementary Table 12**), suggesting a strong binding capacity between these genes and Selumetinib (**Figures 8I–K**). This indicates that IFNG, PTPN6, and SOCS1 may be associated with better therapeutic outcomes of Selumetinib.

### 3.8 Mechanism exploration: the mechanisms of action of IFNG, PTPN6, SLC38A1, and SOCS1 in SKCM

In summary, as key genes among CFRGs, IFNG, PTPN6, SLC38A1, and SOCS1 may be associated with the migration and immunity of melanoma. The researchers subsequently investigated the correlations(**Supplementary Figure S5A**) and PPI relationships (**Supplementary Figure S5B**) of IFNG, PTPN6, SLC38A1, and SOCS1. The results showed that these four genes were positively correlated with each other and exhibited interactions, indicating that they may exert synergistic effects. Combined with the aforementioned research findings, the researchers hypothesize that IFNG, PTPN6, SLC38A1, and SOCS1 may mediate ferroptosis as a complex and activate the PI3K/AKT pathway, thereby affecting the activation of CD8+ T cells and the release of IL10 in TME(**Supplementary Figure S5C**). This leads to the dysregulation of the SKCM immune microenvironment, ultimately resulting in poor prognosis and suboptimal immunotherapeutic efficacy in SKCM patients.

## 4 Discussion

SKCM remains a major global public health challenge. Although immunotherapy and targeted therapies have reduced overall mortality among patients with SKCM, early metastasis continues to drive increasing incidence and mortality in high-risk populations, underscoring the critical limitations of current treatments (2). As a central therapeutic approach, the efficacy of immunotherapy is closely linked to tumor immunogenomic characteristics; however, reliable biomarkers for predicting treatment response are still lacking. This gap hampers precise clinical application and poses significant challenges for patient management.

Progress has been made in the identification of prognostic biomarkers for SKCM. For example, specific circulating DNA markers, such as BRAF and NRAS mutations, have been associated with poor prognosis (28). Eddy C. Hsueh et al. analyzed 31 key genes (including BAP1, MGP, and SPP1) derived from cutaneous and uveal melanoma and established an independent prognostic molecular model using retrospective and prospective datasets (29). Nevertheless, research aimed at improving immunotherapy response and long-term prognosis in SKCM remains limited. There is an urgent need for comprehensive studies to identify novel molecular markers capable of simultaneously predicting therapeutic targets, immunotherapy response, and drug sensitivity. The successful identification of such markers would substantially advance SKCM prevention, treatment, and prognostic evaluation, thereby providing robust evidence to support clinical decision-making.

Research into cell death mechanisms has opened new avenues for the discovery of molecular biomarkers and therapeutic targets, with cuproptosis and ferroptosis emerging as areas of particular interest due to their close involvement in tumor initiation and progression (30, 31). As a copper-dependent form of regulated cell death, cuproptosis exhibits a notable relevance to SKCM. The metabolic dependence of SKCM on mitochondrial respiration, together with elevated copper accumulation in tumor tissues, renders these tumors particularly susceptible to cuproptosis. Consequently, CFRGs have shown promise as prognostic biomarkers, and related predictive models and key genes (such as CDKN2A) have provided new insights into SKCM diagnosis and treatment (32–35).

In contrast, ferroptosis is an iron-dependent form of cell death driven by lipid peroxidation. The high metabolic activity of SKCM generates substantial levels of reactive oxygen species, which can readily trigger ferroptosis. Ferroptosis-related molecules have been shown to predict prognosis and regulate tumor progression, making ferroptosis induction a promising therapeutic strategy for SKCM (36–38). Although integrated analyses of CFRGs have enabled prognostic prediction and immunotherapy response assessment in cancers such as breast and liver cancer (39, 40), studies focusing on CFRGs in SKCM remain scarce. Existing models have not adequately addressed the relationship between cuproptosis and SKCM, and the combined mechanisms of ferroptosis and cuproptosis, as well as their systematic predictive value for the immune microenvironment and clinical prognosis of SKCM, remain unclear (41). Given the pronounced heterogeneity and complex TME of SKCM, this study aims to address these gaps by, for the first time, integrating ferroptosis- and cuproptosis-related mechanisms. We systematically analyze the associations between core CFRGs and patient survival, immunotherapy response, and drug sensitivity, and construct a predictive model aimed at providing novel theoretical evidence and potential therapeutic targets for the precise diagnosis and treatment of SKCM.

Meanwhile, significant breakthroughs have been achieved with the emergence of ICIs in cancer immunotherapy. These agents reactivate T cell-mediated antitumor responses by blocking immunosuppressive signaling pathways, such as PD-1/PD-L1 and CTLA-4. ICIs have demonstrated notable survival benefits in various metastatic cancers (32, 39). In SKCM, combination therapy with nivolumab and ipilimumab has shown substantial clinical benefit in patients with advanced disease, achieving a median overall survival of up to 72 months (40). However, as the clinical use of ICIs has expanded, their limitations have become increasingly evident. IrAEs and drug resistance are now recognized as major factors limiting ICI efficacy. Some irAEs may result in irreversible long-term tissue damage, and combining ICIs with other therapeutic modalities can induce novel or more complex irAE patterns, further complicating clinical management (42).

TME forms a complex and multifunctional system that plays important roles in tumor development, immune evasion, and responses to immunotherapy. It therefore offers promising avenues for overcoming challenges associated with ICI treatment (43). Maibach et al. demonstrated that specific TME cellular subpopulations, such as tumor-infiltrating lymphocytes in SKCM, are correlated with patient prognosis and can also predict responses to ICI therapy, highlighting TME characteristics as valuable indicators for clinical evaluation (44). In this study, we CFRG features with TME analysis to develop a model capable of reliably predicting immunotherapy response and prognosis in SKCM. Using a combination of analytical methods, including WGCNA, ESTIMATE algorithms, and machine learning, we identified PTPN6, SOCS1, SLC38A1, and IFNG from relevant gene modules. Bioinformatics analyses and experimental validation confirmed that these genes are strongly associated with patient survival, immune regulation, and metastatic potential in SKCM. Their expression levels were closely linked to T cell infiltration, with increased expression of PTPN6, IFNG, and SLC38A1 particularly associated with increased CD4^+^ T cell infiltration. In addition, these genes showed significant correlations with immune checkpoint molecules CD274 and CD276. Collectively, our findings indicate that these genes play important roles in TME regulation, immune evasion, and tumor metastasis, thereby directly influencing SKCM development and progression.

IFNG is primarily produced by activated T cells and NK cells and plays a dual role in tumor immunity. On one hand, IFNG acts as a tumor suppressor involved in immunoediting and immunosurveillance processes (45); on the other hand, it can promote SKCM progression and metastasis by reshaping immune cell composition within the TME (46, 47). The IFNG-responsive subtype PSMB9 has been identified as a predictive marker for SKCM prognosis and immunotherapy response (48). Moreover, IFNG can upregulate immunosuppressive molecules, such as IDO1, within the TME (49), thereby influencing tumor survival and progression through modulation of immune signaling dynamics. PTPN6 also shows dual regulatory functions in the TME across various cancers, including glioblastoma, sarcoma, and SKCM. This gene can promote tumor progression through metabolic reprogramming while facilitating immune evasion (50, 51). Conversely, enhanced immunotherapy responses have been observed with PTPN6 through strengthened T cell activation and infiltration (52). Studies further indicate that PTPN6 plays a critical role in suppressing activation-induced cell death in CD4^+^ T cells and modulating PD-1-mediated immunotherapy (53, 54), highlighting its potential as both a prognostic biomarker and therapeutic target in SKCM (55). SLC38A1 is expressed at significantly higher levels in SKCM tissues than in normal epidermal tissues, and suppression of its expression can effectively delay tumor progression, suggesting its potential as a therapeutic target (56). SOCS1 plays a multifaceted role in SKCM. It can function as a tumor suppressor by inhibiting cell mitosis (57), yet it may also suppress antitumor immunity by promoting epithelial–mesenchymal transition and upregulating PD-L1 expression (58). SOCS1 silencing activates the STAT3 signaling pathway, thereby promoting brain metastasis in SKCM (59). In contrast, SOCS1 deficiency in monocytes enhances dendritic cell antigen presentation and antigen-specific antitumor immune responses rather than merely inducing tumor-associated inflammation (60, 61).

Based on the functional characteristics of these key genes, we further used molecular docking analyses to identify potential therapeutic compounds. Among the candidates, selumetinib emerged as a promising option. It is a potent and selective inhibitor of MEK1 and MEK2, with a well-established antitumor mechanism. By inhibiting MEK1/2 activity, selumetinib blocks the MAPK signaling pathway, which is critically involved in tumor cell proliferation, differentiation, and survival (62–64). Currently, selumetinib is primarily approved for the treatment of neurofibromatosis type 1 in children aged 2 years and older with symptomatic, inoperable plexiform neurofibromas (65, 66), and it has also shown activity in multiple myeloma (67). Increasing evidence suggests that selumetinib holds considerable promise for treating advanced SKCM (68, 69). Our findings further indicate that selumetinib has unique potential as a targeted therapeutic agent for SKCM by selectively inhibiting key signaling pathways. This drug may help overcome major treatment challenges, including drug resistance and immune evasion, thereby offering novel directions for improving patient outcomes and developing effective combination therapies.

In summary, IFNG, PTPN6, SLC38A1, and SOCS1 play important roles in modulating the TME and can substantially influence immunotherapy outcomes in SKCM patients. However, current investigations into the interactions between CFRGs and TME in SKCM are largely based on bioinformatic analyses. The precise molecular mechanisms underlying these interactions require further exploration through *in vitro* and *in vivo* studies, and the reliability of associated predictive markers must be validated in multicenter clinical cohorts. Future systematic investigations are expected to uncover key molecular pathways governing CFRGs-TME interactions, thereby providing a more solid theoretical foundation and identifying potential therapeutic targets for the precise diagnosis and treatment of SKCM, ultimately facilitating the translation of these findings into clinical practice.

## Data Availability

The datasets presented in this study can be found in online repositories. The names of the repository/repositories and accession number(s) can be found below: http://xena.ucsc.edu/, http://xena.ucsc.edu/, https://www.ncbi.nlm.nih.gov/geo/, https://www.ncbi.nlm.nih.gov/geo/https://www.proteinatlas.org/, https://www.proteinatlas.org/.

## Supplementary material

Supplementary Figure 1 PCA analysis of CFRGs risk scores. **(A)**PCA principal component score plot of the CFRGs risk scoring model, showing the distribution of data from the low-risk and high-risk groups on PC1 and PC2.

Supplementary Figure 2 Immune infiltration analysis using the xCELL algorithm and ImmuCellAI algorithm. **(A, B)** The ImmuCellAI algorithm and xCELL algorithm show the distribution of immune cell infiltration proportions in the high-risk group (blue) and low-risk group (yellow).

Supplementary Figure 3 Comparison of the relative expression levels of TIDE and Dysfunction. **(A, B)** The differences in the relative expression levels of TIDE and Dysfunction between the high-risk and low-risk score groups.

Supplementary Figure 4 Quality control and preliminary analysis of single-cell RNA sequencing (scRNA-seq) data. **(A)**Gene loading heatmap of the first four principal components (PC1-PC4) in PCA.**(B)**Distribution of the number of unique molecular identifiers (nCount_RNA) detected per cell.**(C)**Distribution of the number of genes (nFeature_RNA) detected per cell.**(D)**Distribution of single and doublet cells in low-dimensional space.**(E)**Cells colored by their inferred cell cycle phases (G1 phase, S phase, G2/M phase), showing cell cycle heterogeneity.

Supplementary Figure 5 Mechanistic exploration of core genes. **(A)**Correlation analysis of core genes.**(B)**PPI analysis of core genes.**(C)**The IFNG/PTPN6/SLC38A1/SOCS1 complex mediates ferroptosis activation and regulates the PI3K/AKT pathway, thereby affecting CD8+ T cell activation and IL-10 secretion in TME.
