# Supplementary figures and images for: Integrating cuproptosis- and ferroptosis-related gene signatures to predict prognosis, immunotherapy response, and drug sensitivity in patients with skin cutaneous melanoma
Source: Front Immunol. 2026 Jan 14;16:1742614. doi: 10.3389/fimmu.2025.1742614 (PMC12847304; doi:10.3389/fimmu.2025.1742614)

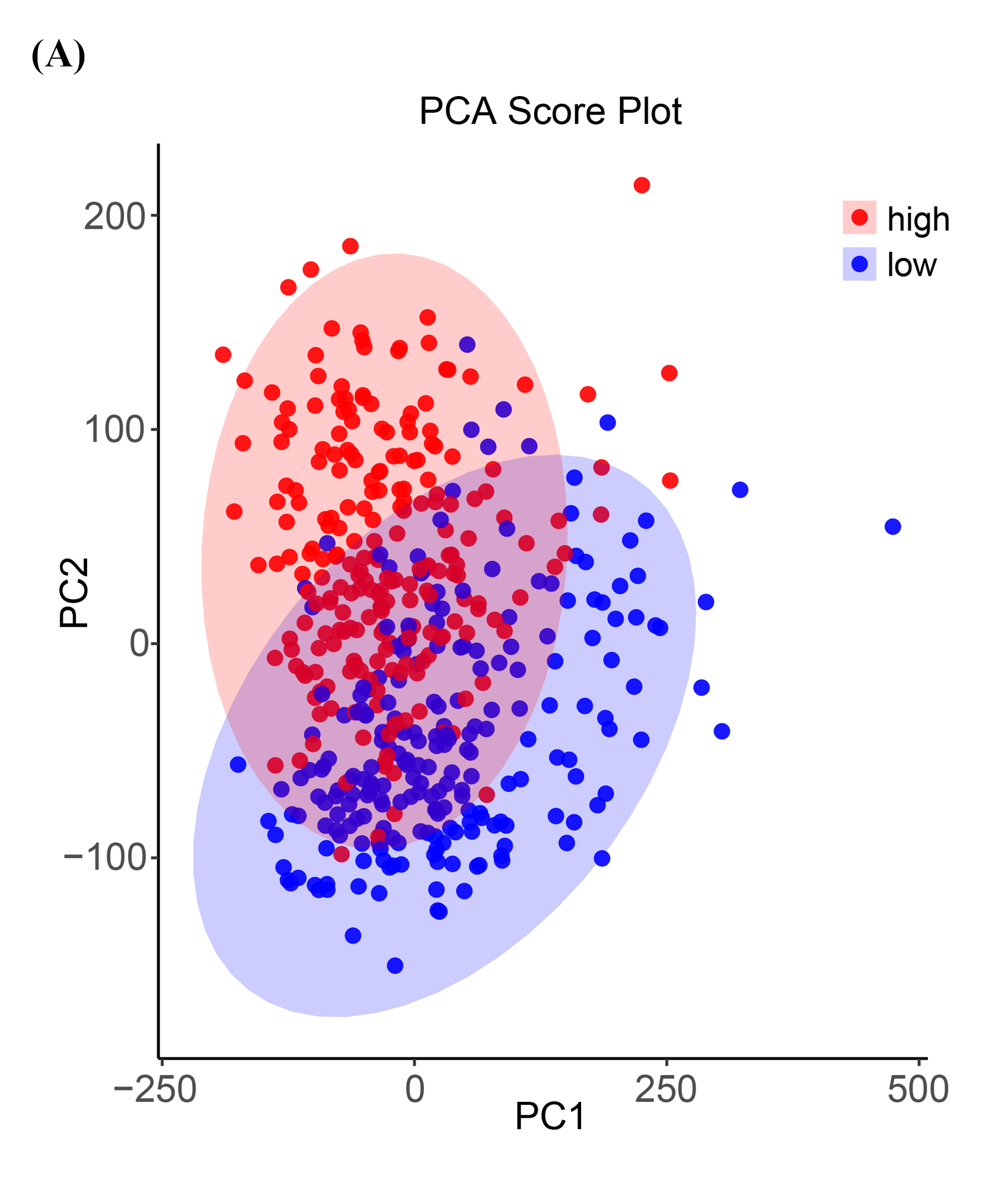

Supplement: Supplementary Figure 1 — PCA analysis of CFRGs risk scores. (A)PCA principal component score plot of the CFRGs risk scoring model, showing the distribution of data from the low-risk and high-risk groups on PC1 and PC2. [file Image1.jpeg]

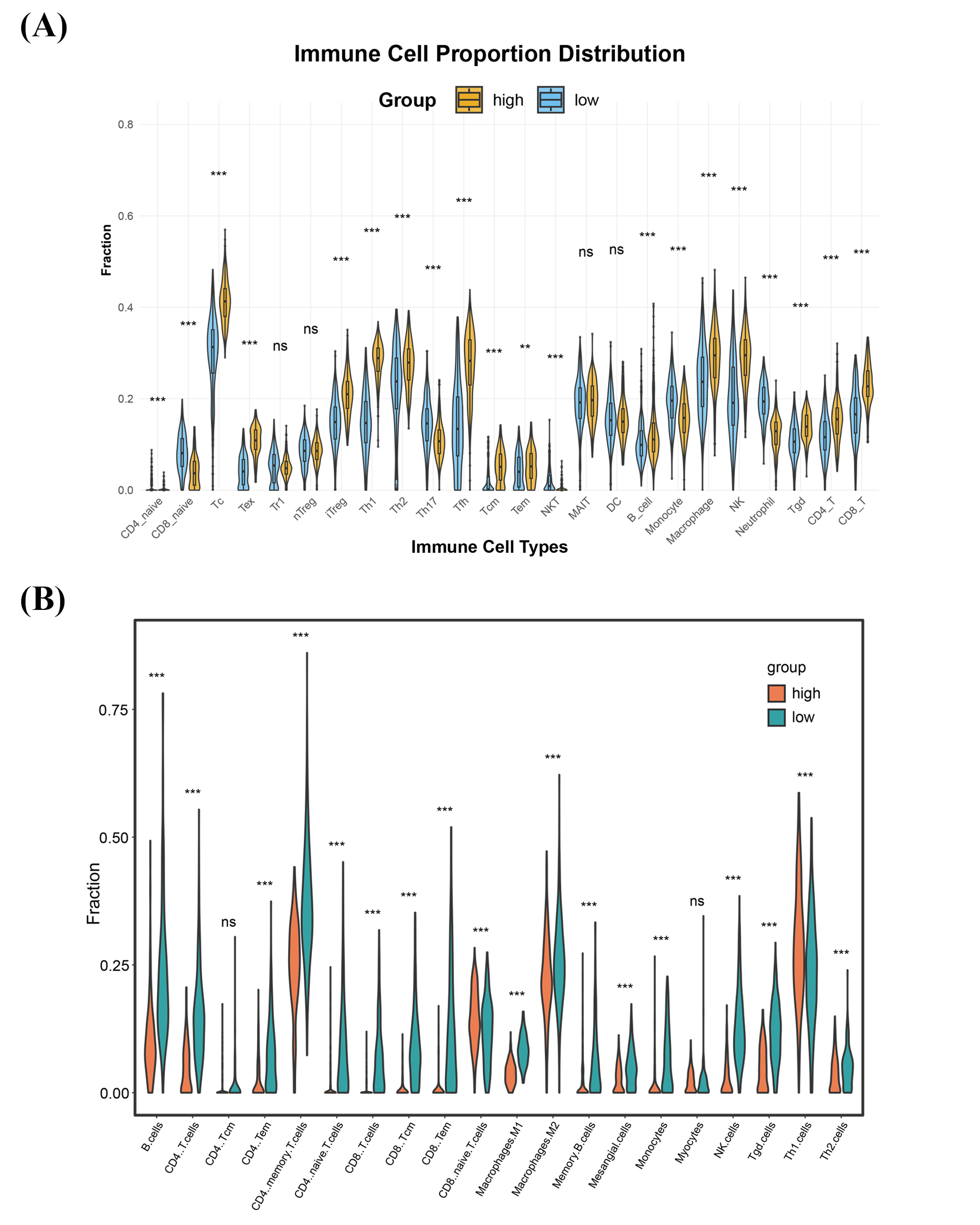

Supplement: Supplementary Figure 2 — Immune infiltration analysis using the xCELL algorithm and ImmuCellAI algorithm. (A, B) The ImmuCellAI algorithm and xCELL algorithm show the distribution of immune cell infiltration proportions in the high-risk group (blue) and low-risk group (yellow). [file Image2.jpeg]

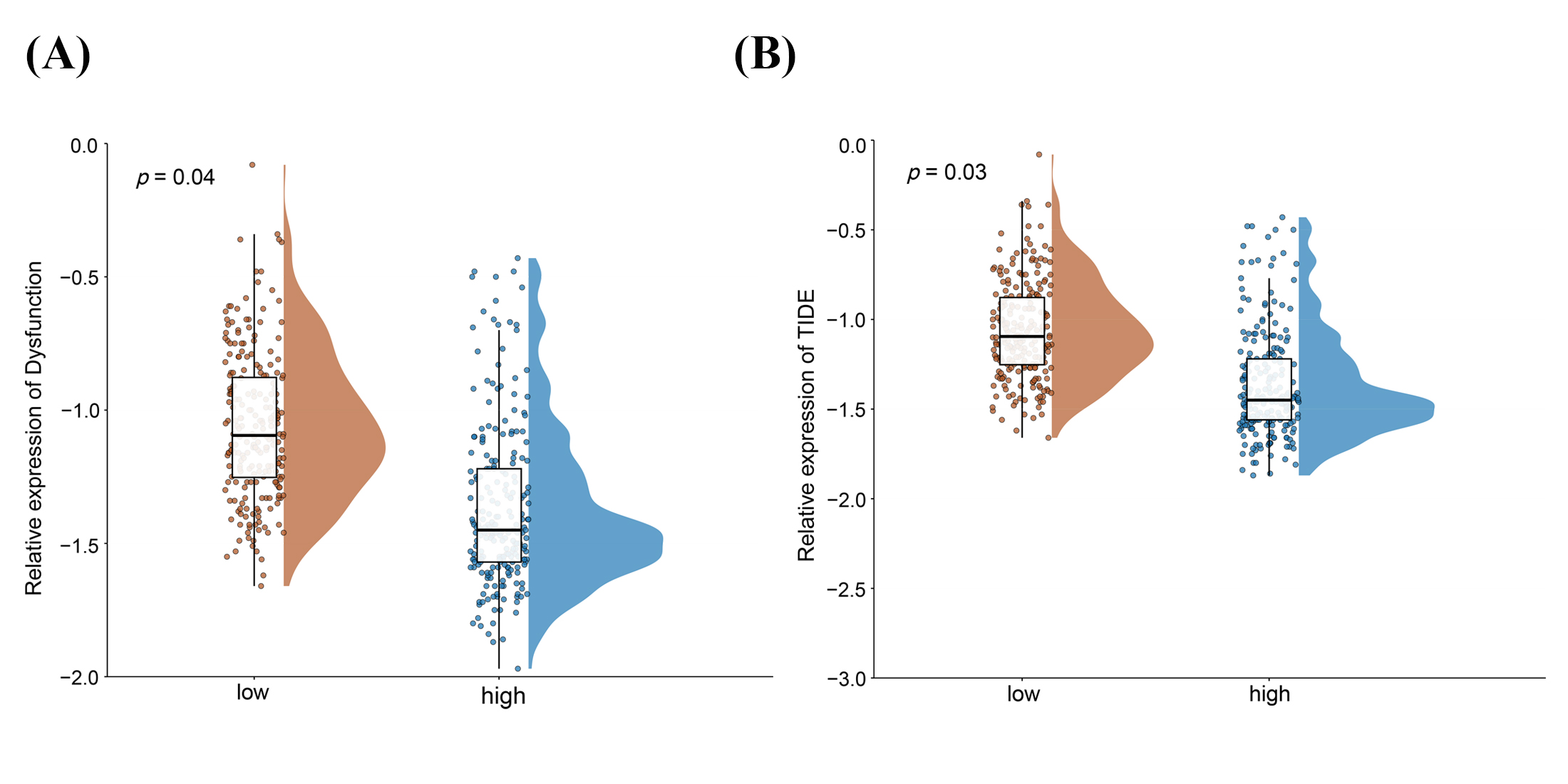

Supplement: Supplementary Figure 3 — Comparison of the relative expression levels of TIDE and Dysfunction. (A, B) The differences in the relative expression levels of TIDE and Dysfunction between the high-risk and low-risk score groups. [file Image3.jpeg]

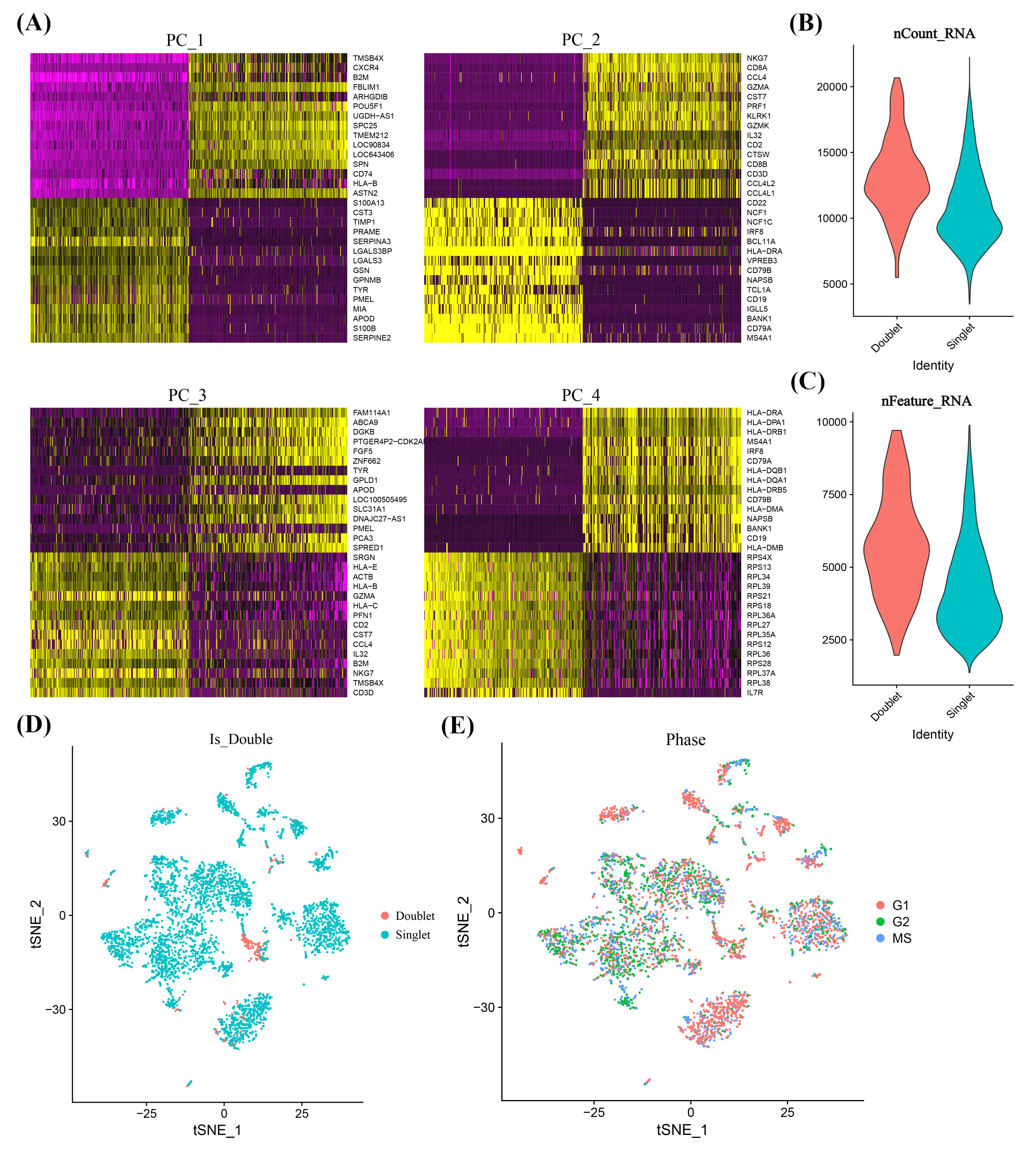

Supplement: Supplementary Figure 4 — Quality control and preliminary analysis of single-cell RNA sequencing (scRNA-seq) data. (A)Gene loading heatmap of the first four principal components (PC1-PC4) in PCA.(B)Distribution of the number of unique molecular identifiers (nCount_RNA) detected per cell.(C)Distribution of the number of genes (nFeature_RNA) detected per cell.(D)Distribution of single and doublet cells in low-dimensional space.(E)Cells colored by their inferred cell cycle phases (G1 phase, S phase, G2/M phase), showing cell cycle heterogeneity. [file Image4.jpeg]
